# The Effect of Commiphora molmol (Myrrh) in Treatment of Trichomoniasis vaginalis infection

**Published:** 2011-07-01

**Authors:** G M El-Sherbiny, E T el Sherbiny

**Affiliations:** 1 Department of Parasitology, Faculty of Pharmacy, October 6 University, Cairo, Egypt; 2 Department of Zoology, El Nahda University, Beni Sweif, Egypt

**Keywords:** Punica granatum, Commiphora molmol, Trichomonas vaginalis, Trichomoniasis, Treatment

## Abstract

**Background:**

Trichomoniasis vaginalis is now an important worldwide health problem. Metronidazole has so far been used in treatment, but the metronidazole-resistant strains and unpleasant adverse effects have been de-veloped. Myrrh is one of the oldest known medicinal plants used by the ancient Egyptians for medical purposes and for mummification. Commiphora molmol (Myrrh) proved safe for male reproductive organ which is the main habitat of T. vaginalis and this study aims to evaluate the efficacy of the herbal against T. vaginalis in females.

**Methods:**

In the present study, 33 metronidazole-resistant T. vaginalis females were treated with a combined course of metronidazole and tinidazole. Those still resistant to the combined treatment were given C. molmol. Also, natural plant extract purified from pomegranate (Punica granatum, Roman) was in-vitro investigated for its efficacy against T. vaginalis on Diamond media.

**Results:**

The anti-T. vaginalis activity of both P. granatum (in-vitro) and C. molmol (in-vivo) extracts gave promis-ing results.

**Conclusion:**

The anti-T. vaginalis activity of P. granatum and C. molmol showed promising results indicating to sources of new anti-Ttrichomonas agents.

## Introduction

Although Trichomonas vaginalis was first described by Donne' in 1836, research on this organism did not begin until the 20th century. The research has been a progression of phases throughout the last 60 years and has gone from developing axenic culture and defining nutritional requirements to finding an effective treatment.[1] It was considered either a harmless vaginal colonizer or simply a minor nuisance.[2] Trichomoniasis was accounted to about half of all the curable sexually transmitted diseases worldwide.[3]The incidence of this sexually transmitted parasite has reached the epidemic levels in many countries.[4] Also, T. vaginalis survived in swimming pool, where human may acquire infection.[5]In USA, annual inci-dence of T. vaginalis reached 5 millions.[6] The general annual adult infection was 180-200 millions and being higher than that of gonorrhea, syphilis, and Chlamydia infections all together.[7] In many Arab countries, trichomoniasis was reported including Jordan,[8]Iraq,[9] Egypt,[10] Saudi Arabia,[11] Libya,[12] and Tunisia.[13] The wide diversion in subtypes of T. vaginalis isolates caused different clinical symptoms with diversity of innate immune responses.[14] The infection was always associated with other sexually-transmitted diseases (STDs) and a sensitive marker for high risk sexual behaviour.[15] T. vaginalis in males caused non-gonococcal urethritis,[16] but with serious complica-tions.[17] Also, T. vaginalis adherence was shown to mediate different gene expressions in human epithelial cells.[18] The premature rupture of membranes, lowbirth weight, preterm labor,[19] female infertility,[20] and postpartum infection, even in asymptomatic women were associated with trichomoniasis.[21] T. vaginalis is a factor in genesis and cause of cervical neoplasia,[22]

and progression of cervical carcinoma,[23] also phago-cytes sperm cells.[24] Unlike other STDs, T. vaginalis rate was more prevalent among women of all ages,[25] and half of them were asymptomatic[26] since trichomoniasis was a curable infection by a single dose metronidazole,[27] successful control of STDs was aided by sensitive, simple and rapid test(s). No doubt, treating patients lowered the overall disease prevalence and morbidity.[28] Metronidazole has so far been the most widely used drug for treating T. vaginalis,[29]but, metronidazole can lead to drug resistance and potential risks of mutagenesis and carcinogenicity.[30] In addition, its side effects such as headache, dry mouth, glossitis, and urticaria caused by lenity treatment or high doses have been described.[31] But, at least 5% of clinical trichomoniasis is caused by strains resistant to commonly used drugs.[32] Also, Hussien et al.[32] reported the presence of different strains of T. vaginalis. The lack of approved alternative therapies for T. vaginalis treatment means that higher and sometimes toxic doses of metronidazole were used.[33] Tinidazole (Fasigyn), a second-nitronidazole generation has shown to be an effective therapy in metronidazole-resistant T. vaginalis, with several advantages over metronidazole including greater in vitro potency against both sensitive and resistant strains of T. vaginalis, a more prolonged duration and improved patient tolerability.[34] Cross-resistance among mitronidazole doses occurred, and thus metronidazole resistant strain was treated with tinidazole but rapid development of tinidazole-resistant T. vaginalis due to the similarities of metabolic pathway of both.[4]

According to world health organization (WHO), more than 80% of the world's population relies on traditional medicine for their primary healthcare needs. Use of herbal medicines represents a long history of human interactions with the environment. Plants used for traditional medicine contain a wide range of substances that can be used to treat chronic as well as infectious diseases.35 The medical value of plants lies in some chemical substances that produce a definite physiological action on the human body. The most important of these bioactive compounds of plants are alkaloids, flavanoids, tannins, and phenolic compounds.36

Natural products are not only the basis for traditional or ethnic medicine, but also screening natural plant products provided highly successful new regimens for human welfare.37 Many new natural product groups have revealed anti-parasitic properties of surprising efficacy and selectivity.38 In the present study, Mirazid was given to metronidazole and tinidazole resistant T. vaginalis infected women. Also, the efficacy of Punica granatum extract against cultured T. vaginalis was evaluated.

## Materials and Methods

The institutional review board of hospital approved this study. The study was registered at the Ministry of Scientific Research Academy of Scientific Research and Technology (292473).

We informed women to allow for an attrition rate (i.e. women who discontinue participation in the study entirely, including failure to complete all follow-up). Thus, [33] women were available to be studied.

The patients were recruited from hospitals of Cairo Curative Organization of Egypt. Potentially eligible patients were identified through hospitals registries for a 3-years retrospective inclusion, and through treating physicians, for the 2 years prospective inclusion (Patient who met the case definition of metronidazole resistant vaginal trichomoniasis were selected). A letter was sent for them while had been signed by their physician informing them of the study protocol and, if initially interested, asking them to complete a brief questionnaire to screen for trichomoniasis symptoms. Women who were assigned into control group were given the choice of undergoing the study program.

Metronidazole- resistant trichomoniasis was defined clinically as failure to respond to conventional therapy with oral metronidazole, 500 mg for 7 days (Total dose, 7 g). Patients in whom treatment failed and for whom re-infection from a sexual partner was a possibility were excluded from the study. "Failure to respond" was defined as persistence or recurrence (with 28 days) of symptoms and signs of vaginitis together with the following confirmatory laboratory feature of vaginal trichomoniasis: high vaginal pH, increased numbers of polymorphonclear leukocytes, and a visualization of motile trichomonads using microscopy. In vitro Trichomonas cultures of individual specimens were performed using Dimoned's medium Modified (Remel), and they were examined at 24 hr and 48 h for the presence of motile trichomonads.

Two vaginal swabs were obtained from the childbearing period trichomoniasis infected women by sterile vaginal swab. The first swab was obtained from the lateral wall of vagina and was used to make

a wet mount preparation on a glass slide with a drop of normal saline and looking for motile trichomonads.39 The second swab was obtained from the posterior fornix of the vagina and inoculated immediately after collection in Diamond media at 32oC and examined for motile trichomonades at 24, 48, and 96 hr of incubation.[40]The efficacy of metronidazole and tindazole was evaluated in 33 patients. Both drugs were given orally in a single dose of 2 g for a minimum of 3 days or extended as indicated. A combination of vaginal metronidazole and oral tinidazole was tried. The effectiveness of an oleo-resin extract derived from Myrrh, Commiphora molmol (Mirazid) was given to the metronidazole and tinidazole resistant females as two capsules (600 mg) for six to eight successive days on an empty stomach two hours before breakfast. All patients were seen immediately after treatment completion and again 4 to 6 week later. Patients were considered cured if all symptoms and signs of vaginal trichomoniasis resolved with therapy and the patients had negative results of microscopy at least 4 weeks after completion of therapy. In the majority of patients, a follow- up culture was performed. Patients were also instructed to notify investigators if symptoms returned after the final follow-up visit. Follow up was done both clinically and parasitologically by the examination of vaginal discharge as wet mount smear and culture on Diamond media.

**Figure 1:**
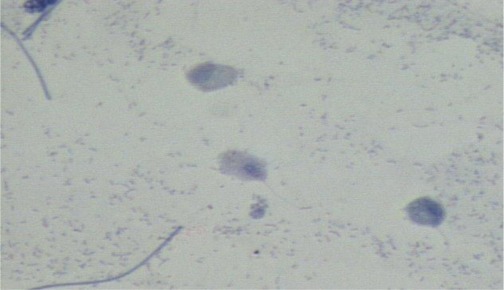
Normal T. vaginalis stained by ZN

**Figure 2:**
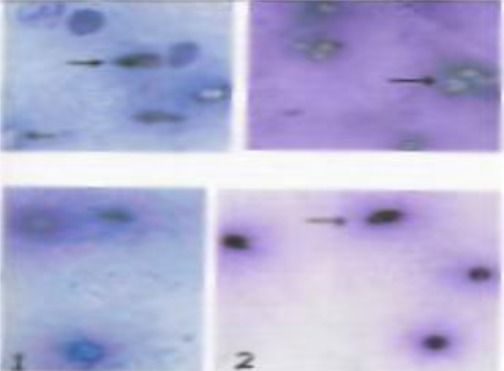
ZN stained smear showing lethal effect of P. granatum on T. Vaginalis

Punica granatum fruits were selected from tree that was neither treated with any insecticide nor with plant fertilizer. The fruits were carefully washed with sterile distilled water. The peels of pomegranate fruits were manually removed, sun dried and powdered. Powder was extracted with a Soxhlet extractor using methanol for 24 hr.[41] Extract was filtered through a Whatman filter, No. 41 filter paper, for removal of peel particles (Solvent was removed under gentle pressure in rotary evaporator till dryness, and residue was stored at 4oC).[42] Extract was tested at different concentrations, diluted with sterile normal saline against cultured T. vaginalis. Control culture lacked extract. All culture-media were incubated at 37oC and examined for living T. vaginalis (Figure 1 and Figure 2). The results are expressed as means and values were evaluated by the Chi Square test and p<0.5 was considered significant.

## Results

Review of medical records revealed 33 cases of met-ronidazole- resistant trichomoniasis seen during a 3 years period (2005-2008). The mean age of the female patient was 37.2 years (range= 25-58 years). Duration of vulvovaginal symptoms was from 4 months to 5 years. Seventy five percent of referred women had previously diagnosed resistant trichomoniasis, and 25% of the women were referred because of refractory vaginitis of unknown aetiology. Before referral, all patients had received and did not respond to multiple courses of oral metronidazole.

Fifty percent of patients (9/18) given oral tinidazole were cured and 73% of patients (11/15) given a combination of oral metronidazole and vaginal tinidazole were treated(Table 1) . Besides, This figure for patients who did not respond to combination of metronidazole and tinidazole oral or vaginal and were cured by Mirazid was 84.6% (11/13). In vitro susceptibility of isolates of T. vaginalis to P. granatum extract was determined. At pH=4.65, P. granatum showed a therapeutic effect against T. vaginalis, the organism was dead immediately in the

tube containing 50 mg and100 mg of the extract, and within 0.5 hour in the tube with 20 mg extract. At pH=6.00, however, P. granatum extract had no effect against the organism.

**Table 1:** Trichomoniasis cases treated by metronidazole and tinidazole.

**Drug used**	**Total**	**Cure**	**Not**	**%Cure**
Tinidazole	18	9	9	50
Oral metronidazole + vaginal tinidazole	15	11	4	73
Total	33	20	13	60.6

## Discussion

There is a need for studies into other chemicals and/or medicinal plants or herbs for alternative regimens against T. vaginalis to be inexpensive, effective, safe to use and with short course of treatment. Globally, herbal remedies have been studied under rigorous controls and was technologically approved by authors in many countries.

Factors that predispose women to vulvovaginitis candidiasis and trichomoniasis are pregnancy, diabe-tes, HIV infection, higher dosage of oral antibiotics or corticosteroid use, immunosuppression, more patho-genic normal flora, and history of recurrent infection. Other factors that may increase the incidence include the use of perfumed feminine hygienic sprays, tropi-cal antimicrobial agents, and tight poorly ventilated clothing and under wear.[43]

Successful determination of biologically active compounds from plant material is largely dependant on the type of solvent used in the extraction proce-dure, properties of a good solvent in plant extraction that induces ease of evaporation at low heat, promo-tion of rapid physiologic absorption of the extract, a preservative action and inability to cause the extract to complex or dissociate.[44] The choice will also de-pend on targeted compounds. The most commonly used solvents for investigation of microbial activity in plants are methanol, ethanol, and water.[45] In this study methanol extract was used.

In the present study, P. granatum extract on T. vaginalis in Diamond media showed 100% efficacy in dilution up to 10%. On the other hand, extracts in the concentrations of 5%, 1% and 0.5% killed 40%, 25% and 10% of T. vaginalis respectively.

Metronidazole has a worldwide use within the last 2 years of its introduction, but the lack of surveillance data of vaginal trichomoniasis and clinical and mi-crobiological response to treatment, incidence of met-ronidazole resistance has spared. Lossick and Kent46 found that the high level resistance to metronidazole occurred in one out of 2000-3000 cases of vaginal trichomoniasis cases. Saurina et al.[47] studied the prevalence of in vitro metronidazole resistance among outpatients attended urban clinic, found that 3/118 (2.5%) of T. vaginalis isolates from 107 patients ex-hibited aerobic low level resistance. The development of drug resistance in human against commonly used treatments has necessitated a search for new anti-agent substances from other sources including plants.[48] Myrrh is an oleo-gum resin obtained from the stem of the herbal tree Commiphora molmol. It contains a resin (Myrrhin) which is a volatile oil (Myrrh), gum and a bitter principle.[49] Myrrh was used by Sumerians and Greeks to treat worms, stomach pain, flatulence particularly in children,[50] anti-inflammatory, anti-ulcer, anti-mutagenic, anti-cytotoxic, anti-carcinogenic,[51] and anti-diabetic prop-erties.[52] Also, C. myrrha and various other species of Commiphora are recognized to possess significant antiseptic, anesthetic, and anti-tumor properties.[53] Its safety and effectiveness was proved in the treatment of human schistosmoiasis,[54][55][56] fascioliasis of human,[57] and animals,[58] moniziasis,[59] strongyloidiasis,[60] heter-ophyiasis,[61] and both species of hymenolepiasis.[62] Also, myrrh has larvicidal action against larvae of both Culex pipiens and Aedes caspius,[54] molluscicidal action against Biomphalaria alexandrina, Bulinus truncatus and Lymnaea cailliaudi,[63] Bithynia connol-lyi, the snail vector of the trematod parasite Opisthor-chis sp.,[64] and Lymnaea natalensis.[65] C. molmol proved safe for male reproductive organs which is the main habitat of T. vaginalis.[66] Omar et al.[67] tested the safety of mirazid on adult male albino rats by assess-ment of serum levels of ALT, AST and bilirubin and histopathology of liver. They found a non-significant increase in these enzymes and bilirubin levels. Auffray[68] in France stated that essential oil of C. myrrha ha the best protection against squalene per oxidation, and that

sun care cosmetics should make use not only of free radical scavengers but also of singlet oxygen quenchers.

The Pomegranate fruit has been used for centuries in ancient cultures for its medicinal purposes. It is widely consumed fresh and in beverage forms as juice and wine.[69] Properties attributed to its high con-tent of polyphenols, including ellagic acid in its free and bound forms, and other flavouroids.[70] In the last two decades, many authors dealt with P. granatum (Pomegranate) as a medicinal plant.[71] It is a shrub or small tree which several parts have been used by old Indian physicians. Nowadays, parts of pomegranate are used as an astringent, anti-microbial hemostatic, anti-diabetes, anti-helminthes,[72] anti-prostate cancer,[73] improved anti-oxidant function in elderly subjects,[74] anti-fungal peptide,[75] and anti-Candida,[76] mouth-anti-T. Gingivalis,[77] as heart-healthy juice,[78] and preven-tion of the cardiovascular diseases.[79] Dried per carp was decocted with other herbs and used to treat colic, dysentery, leucorrhoea,[80] and as larvicide against my-iasis producing larvae of Lucilia sericata.[81] Also, the rind of fruit and flower, combined with aromatics, such as cloves, cinnamon, coriander, pepper etc as bowel astringent in the diarrhea.[82] It was used exter-nally in treatment of the vaginal discharge, mouth sores, and throat infections.[83] Methanol extracts of P. granatum fruit exhibited a higher degree of antimi-crobial activity.[84] The fruit was successfully used to treat dysentery, diarrhea and gastralgia.[85] Voraavu Thikunchai et al.[86] reported that P. granatum contains 25% tannins which made it an effective astringent. In old medicine, the pomegranate as a pharmacy unto itself was used as an anti-parasitic agent, a blood ton-ic and to heal apathies and ulcers.[87]

This study shows statistically significant effects on resistant T. vaginalis strains. These proposed benefits, however, are in assays that are as yet invalidated, and further research is needed to prove the validity of these tests. In conclusion, the results in the present study support the two safe plant extracts (Commiphora molmol and Punica granatum) proved to be valuable agents in treating of T. vaginalis infection, and will form the basis for further investigation in the potential discovery of new natural bioactive compounds.
